# Tissue-specific body composition changes and muscle performance decline: a 5-year prospective study

**DOI:** 10.1007/s40520-026-03425-0

**Published:** 2026-06-13

**Authors:** Ling Wang, Yandong Liu, Wenshuang Zhang, Bo Hu, Kangkang Ma, Fengyun Zhou, Zitong Cheng, Qingyu Zhang, Xiaoguang Cheng, Yi Yuan, Giuseppe Guglielmi, Ling Oei

**Affiliations:** 1 Department of Radiology, Beijing Jishuitan Hospital, Capital Medical University, National Center for Orthopedics, Beijing, China; 2 Department of Clinical and Experimental Medicine, University of Foggia, Foggia, Italy; 3 Department of Internal Medicine, Erasmus MC, Rotterdam, Netherlands; 4 JST sarcopenia Research Centre, National Center for Orthopaedics, Beijing Research Institute of Traumatology and Orthopaedics, Beijing Jishuitan Hospital, Capital Medical University, Beijing, China

**Keywords:** Body composition, Muscle strength, Physical performance, Follow-Up Studies, Ageing

## Abstract

**Objective:**

The change of muscle performance plays an important role in sarcopenia. Our objective was to examine longitudinal changes in body composition in older individuals in relation to changes in physical performance.

**Methods:**

120 individuals were recruited from the neighborhood of the hospital. Computed tomography (CT) scans, handgrip strength (HGS) and the Timed Up and Go test (TUG) were obtained at baseline and follow-up after 5 years. Changes in muscle cross-sectional area and muscle density and adipose area were evaluated. Associations of changes in body composition with changes in HGS and TUG were tested in logistic regression models after adjusting for baseline age, height, weight and type 2 diabetes.

**Results:**

Among females, each decrease (per cm^2^) of anterior compartment of thigh muscle size was associated with a 101% decrease of HGS (95% confidence interval [CI], 1.10–3.67; *P* = 0.02) and an 121% longer TUG (95%CI, 1.16–4.24; *P* = 0.02). Among males, each decrease (per HU) of psoas major muscle density (OR, 0.37; *P* = 0.04) would decrease the risk of decreased HGS.

**Conclusions:**

CT-based body composition changes, atrophy of thigh muscles in particular, are associated with loss of muscle strength and physical performance.

**Advances in knowledge:**

Key findings include significant reductions in muscle size and density across multiple compartments (paraspinal, pelvic and thigh muscles), greater adipose tissue increase in females, and sex-specific associations between thigh muscle atrophy and declines in HGS and TUG performance. These results align with prior cross-sectional evidence of age-related sarcopenia but extend them by quantifying longitudinal changes and their functional consequences.

## Introduction

 Age-related declines in skeletal muscle mass and function, coupled with progressive adipose tissue redistribution, are hallmarks of sarcopenia and frailty, contributing significantly to disability, falls, and loss of independence in older adults [1–5]. While cross-sectional studies have established associations between muscle atrophy, ectopic fat accumulation, and functional impairment, longitudinal data remain scarce, particularly regarding tissue-specific trajectories of decline and their sex-specific implications for physical performance [6–8]. Sarcopenia research has traditionally focused on limb muscles, such as the quadriceps, yet emerging evidence suggests that pelvic, paraspinal, and functionally compartmentalized thigh muscles (anterior, medial, posterior) exhibit distinct atrophy patterns tied to their biomechanical roles [9–11]. For example, the anterior thigh compartment, rich in type II fibers critical for power generation, may be more vulnerable to age-related decline than slower-twitch posterior muscles [10]. Similarly, paraspinal and psoas major muscles, essential for spinal stability and posture, show atrophy rates influenced by compensatory mechanisms [12, 13]. However, no prior study has systematically compared longitudinal changes across these muscle subgroups or evaluated their differential associations with functional outcomes in men and women. Body mass index (BMI) remains a widely used but inadequate metric for aging-related body composition shifts, as it fails to distinguish between muscle loss and fat redistribution [14, 15]. Ectopic fat deposition—particularly in visceral (VAA) and intramuscular depots—is increasingly recognized as a driver of metabolic dysfunction and physical decline [14–17]. Studies have used opportunistic computed tomography (CT) scans to investigate the cross-sectional areas of adipose areas, muscle size and muscle density of skeletal muscles captured in the field of view as surrogate for adipose areas and muscle mass [18–20]. Yet, few studies leverage CT’s potential to track longitudinal changes in community-dwelling older populations.

Sex differences further complicate this landscape. Men typically exhibit greater baseline muscle mass but faster age-related declines, while women face higher risks of adiposity-related metabolic complications [21, 22]. Whether these disparities extend to compartment-specific muscle atrophy or ectopic fat accumulation remains unclear, limiting the development of targeted interventions.

In this 5-year longitudinal study of adults aged ≥ 50 years, we aimed to: (1) characterize tissue-specific changes in muscle (size, density) and adipose tissue (subcutaneous, visceral) using serial CT imaging; (2) determine sex-specific associations between these changes and declines in handgrip strength (HGS) and Timed Up and Go (TUG) performance. By addressing these gaps, our work provides critical insights into the mechanisms of functional decline and informs strategies to preserve mobility in aging populations.

## Materials and methods

### Study design and participants

**Fig. 1:**
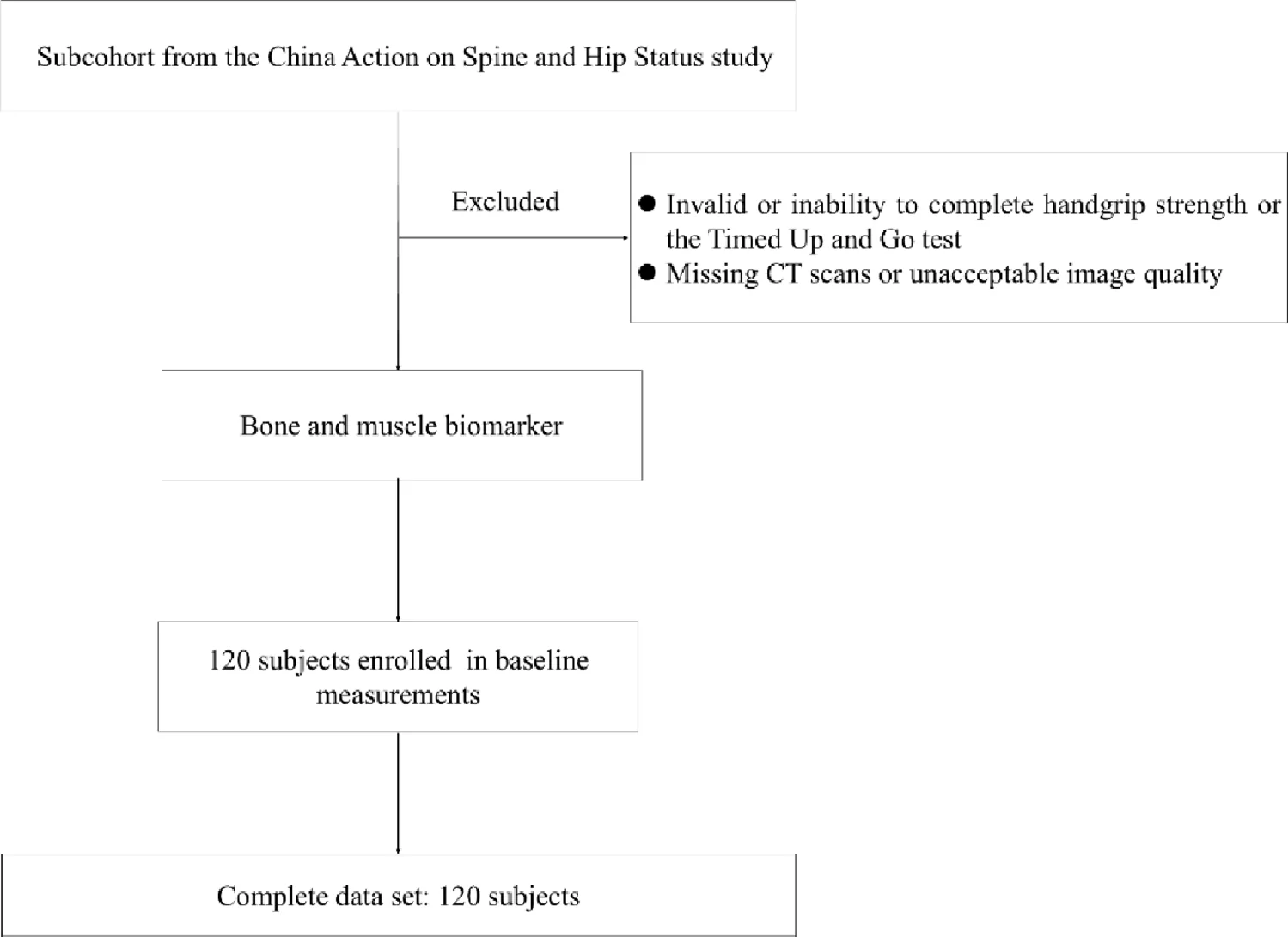
Flow chart of participants selection for the study

The China Action on Spine and Hip Status study enrolled community-dwelling subjects aged 50 years and older from the neighborhood of our hospital [20]. This study included a subcohort from the original participants to search for the musculoskeletal biomarkers. Participants performed quantitative CT (QCT) scans of the lumbar spine, hip, and mid-thigh at baseline (between March 2017 and June 2017) and 5-yr follow-up (between July 2022 and August 2022). Exclusion criteria were inability to complete HGS or TUG tests, missing CT scans or unacceptable image quality. The remaining subjects were 120 cohort members (72 females, 48 males) with acceptable image quality at both baseline and follow-up (Fig. 1). These participants did not take drugs that could influence muscle mass and strength. The median follow-up time was 5 years. The study was approved by the ethics committee of our hospital (approval number No. 201512-02) in accordance with the Declaration of Helsinki and each participant signed informed consent.

### CT scan acquisition

The same Toshiba Aquilion CT scanner (Toshiba Medical Systems Division, Tokyo, Japan) was used to scan at baseline and 5-yr follow-up. CT scans of the lumbar spine CT scans including vertebrae L1-L5 were taken. While participants were lying supine, with their legs extended and their feet secured, a 1-mm thick axial image was taken 15 cm proximal to the top of the patella. The position of this section was determined from a scout view as the center of the long axis of the femur. The hip CT scans were obtained extending from the top of the acetabulum to 3 cm below the lesser trochanter in supine position and included both legs [20]. Scan parameters were 120 kVp, 125 mAs, 50 cm field of view, 512 × 512 matrix, 1-mm reconstructed slice thickness.

**Fig. 2:**
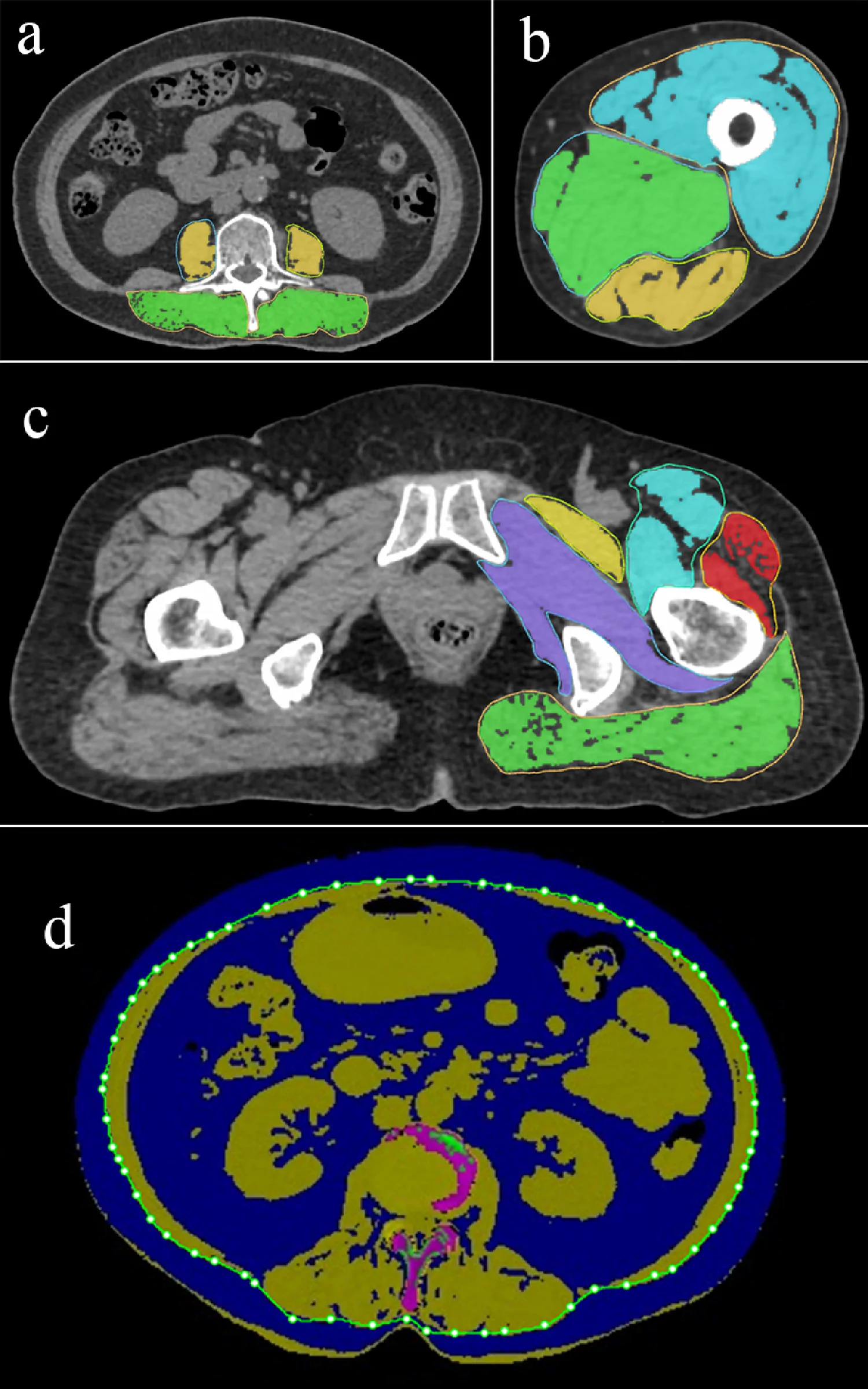
(**a**) Measurement of cross-sectional area and mean computed tomography (CT) values of the paraspinal (green) and psoas major (yellow) at the level of L3; (**b**) Measurement of the anterior compartment (blue), the medial compartment (green) and the posterior compartment (yellow) of thigh muscles at the level of mid-thigh; (**c**) Measurement of extensors (green), external rotators (purple), adductors (yellow), flexors (blue) and abductors (red) at the level of the lesser trochanter; (**d**) Measurements of total adipose area (TAA) and visceral adipose area (VAA) were semi-automatically completed, and green dots show the region of interest demarcating visceral and subcutaneous adipose tissue at the L3/4 level

**Fig. 3:**
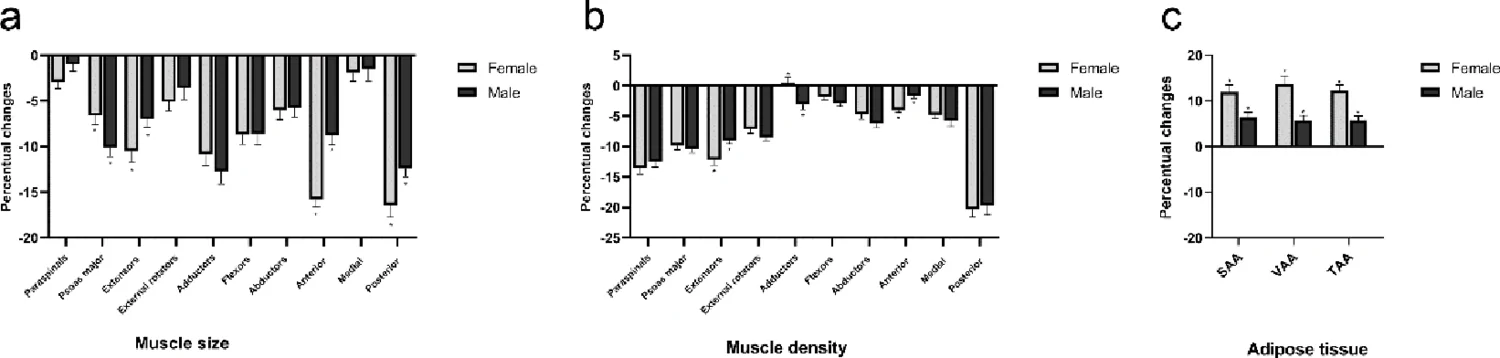
5-yr gender-stratified percent changes in skeletal muscle size (**a**), muscle density (**b**) and adipose tissue (**c**). Anterior, anterior compartment muscles; Medial, medial compartment muscles; Posterior, posterior compartment muscles; SAA, subcutaneous adipose area; VAA, visceral adipose area; TAA, total adipose area

### Measurements of muscle property and adipose tissue

Cross-sectional area (cm^2^) and density (HU) of skeletal muscles were measured on one slice each. At the level of L3, the paraspinal muscles and psoas major muscles were measured. In the hip, the pelvic muscles (extensors, external rotators, adductors, flexors and abductors) at the level of the lesser trochanter were measured. Finally, at the level of mid-thigh, the anterior compartment muscles (sartorius, rectus femoris, vastus lateralis, vastus medialis and vastus intermedius), the medial compartment muscles (adductor brevis, adductor longus, adductor magnus, gracilis, pectineus) and the posterior compartment muscles (biceps femoris, semitendinosus, and semimembranosus) were measured (Fig. 2).

OsiriX software (Lite Version 10.0.2, Pixmeo, Geneva, Switzerland) was used for the muscle measurements. Muscle segmentation was drawn manually by using the “pencil” tool to outline muscle contours. Then the 2-dimensional/3-dimensional segmentation module was applied to semiautomatically select skeletal muscles regions within the preset HU intensity thresholds (-30 to 150 HU). A threshold of -29 HU was applied to segment muscle tissue from fat. Finally, the muscle size and density values were worked out [20].

QCTPro ((Mindways Inc., Austin, TX, USA) software was used for the adipose area measurement. Total adipose area (TAA) and visceral adipose area (VAA) were calculated semiautomatically using the commercial software package: “Tissue Composition Module” Beta 1.0 at the L3/4 level. The subcutaneous adipose area (SAA) was referred as the area of adipose tissue between the skin and the trunk muscles at the L3/4 level. VAA was considered as all intra-abdominal adipose tissue areas within the abdominal cavity of the rectus, external oblique, lumbar quadrate, and psoas muscles (Fig. 2) [19].

To ensure the comparability of measurements for longitudinal analyses, the same levels were selected at baseline and 5-yr follow-up CT images. All the measurements were performed by the same observer who had 4 years of experience in reading muscle imaging. To assess variability, two trained observers evaluated scans for 20 images 2 weeks later. Inter- and intra-observer variability were good (intraclass correlation coefficients > 0.80).

### Muscle strength and physical performance

A Jamar dynamometer (Jamar, Los Angeles, CA, USA) used to analyze HGS of the dominant hand. Three attempts with a 1-minute interval between them were recorded in kilograms, and the maximum value was used for the further analysis. The TUG test was performed by recording the time needed by a subject to rise from an armchair, walk 3 m on a line drawn on the floor, turn, and walk back to the chair to a seated position. Details of the TUG test and measuring HGS were previously described [20].

### Data collection

Demographic variables and health-related data at baseline and follow-up included: age, sex, height, weight, BMI, type 2 diabetes. Other health-related data were retrieved from the patient’s medical file or from the healthy participants’ medical records [20].

### Statistical analysis

SPSS (v.26.0; IBM Corp, Armonk, NY) were used for the analyses. We calculated percent changes scores in body composition, percent changes scores = (follow-up values - baseline values)/ baseline values. Decreases in body composition parameters (i.e. negative change scores [results < 0]) denoted loss of muscle mass and fat, and decreases in HGS represented loss of muscle strength, whereas increases (i.e. positive change scores [results > 0]) in TUG indicated loss of physical performance.

Changes in the demographic variables and body composition from baseline to the 5-yr follow-up were compared using paired t-tests. Percent changes in body composition between females and males were compared using Student t-tests. Post hoc analyses for the muscle main effect (*P* < 0.05) were completed with pairwise comparisons with Bonferroni correction. Age-stratified analyses were carried out, using the group *≤* 70 years as a reference category, to compare the differences in percent changes in body composition in both genders across age categories, respectively. Multi-factor analysis of variance was performed to investigate any possible interaction among the factors. Multi-factor analysis of variance was applied to reveal interactions among the factors (age, gender and type 2 diabetes). Logistic regression models were used to estimate the associations between baseline body composition and (i) baseline HGS and (ii) baseline TUG; between percent changes in body composition and (i) percent changes in HGS and (ii) percent changes in TUG, with adjustments for baseline age, height, weight and type 2 diabetes. We separated the participants into ‘low’ versus ‘high’ groups using the median as a cut-off for analyses for HGS and TUG. The low group represented lower HGS or longer TUG and the high group represented higher HGS or shorter TUG. A *P* < 0.05 was considered statistically significant.

## Results

Participant characteristics of the study cohort are described in Table 1. Seventy-two females and 48 males were enrolled in the study. Of the 12 participants included in this study, 31 females and 11 males had diabetes. Here we describe the longitudinal changes observed during follow-up. During the five-year study follow-up, BMI increased in females, height decreased in both females and males.

Table 2 shows 5-yr longitudinal changes in muscle properties and adipose tissue. Most of the parameters for muscle size and density declined over the 5 years follow-up in both genders (all *P* < 0.01). Fat masses increased over the 5 years (SAA: 133.2 cm^2^ vs. 145.8 cm^2^, *P <* 0.01; VAA: 198.4 cm^2^ vs. 215.9 cm^2^, *P* < 0.01; TAA: 331.1 cm^2^ vs. 361.8 cm^2^, *P* < 0.01). TUG increased (females: 8.0 s vs. 11.4 s; *P* < 0.01; males: 8.1 s vs. 11.9 s; *P* < 0.01) while HGS declined compared with the baseline values (females: 21.7 kg vs. 20.1 kg; *P* < 0.01; males: 34.4 kg vs. 30.9 kg; *P* < 0.01).

Hierarchical patterns of the atrophy in muscle sizes and densities were observed in both genders. In females, the atrophy of muscle densities showed a hierarchical pattern: posterior compartment of thigh > paraspinals and extensors > psoas major > external rotators > medial compartment of thigh, abductors and anterior compartment of thigh > flexors and adductors. But another hierarchical pattern in the magnitude of atrophy was found in muscle size: posterior and anterior compartment of thigh > adductors, extensors and flexors > psoas major, abductors and external rotators > paraspinals and medial compartment of thigh. In males, the atrophy of these muscles in muscle density also demonstrated a hierarchical pattern: posterior compartment of thigh > paraspinals > psoas major, extensors and external rotators > abductors and medial compartment of thigh > adductors, flexors and anterior compartment of thigh, while the hierarchical relationship in the magnitude of atrophy was present in muscle size: adductors and posterior compartment of thigh > psoas major, anterior compartment of thigh and flexors > extensors and abductors > external rotators, medial compartment of thigh and paraspinals.

### Key 5-Year changes in muscle and adipose tissue

Table 3 shows key 5-year changes in muscle and adipose tissue. 5-yr percentual changes in body composition are displayed in Fig. 3. Females demonstrated a greater 5‑year percent decrease in the anterior and posterior compartments of thigh muscle size than males (− 15.8% vs. −8.8% and − 16.4% vs. −12.4%, respectively). However, the 5‑year percent decrease in psoas major muscle size was smaller in females than in males (− 6.6% vs. −10.1%). For muscle density, the 5‑year percent decrease was greater in females than in males in the extensors (− 12.2% vs. −9.0%) and in the posterior compartment (− 20.3% vs. −19.6%). With respect to adipose tissue, the 5‑year percent increase in subcutaneous abdominal adipose area (SAA) and visceral abdominal adipose area (VAA) was larger in females than in males (+ 11.9% vs. +6.4% and + 13.6% vs. +5.7%, respectively). Table A1 shows 5-yr percentual changes in body composition by gender and age categories.

There was a significant interaction between changes in adipose tissue and gender (*P* < 0.05). We did not observe statistically significant interactions between changes in adipose tissue and age (*P* > 0.05), nor between changes in adipose tissue and type 2 diabetes (*P* > 0.05).

**Table 1:** Participant Characteristics (*n* = 120)

Variable	Female (*n* = 72)	Male (*n* = 48)	Total
Age (years)	67.6 → 72.7	69.2 → 74.3	68.3 → 73.4
Height (cm)	158.9 → 158.1*	170.8 → 169.8*	163.6 → 162.8*
BMI (kg/m²)	25.3 → 26.0	24.1 → 24.6	24.8 → 25.4*
TUG (s)	8.0 → 11.4*	8.1 → 11.9*	8.0 → 11.6*
HGS (kg)	21.7 → 20.1*	34.4 → 30.9*	26.8 → 24.5*

*P<0.01 for changes over 5 years. BMI: Body Mass Index; TUG: Timed Up and Go; HGS: Handgrip Strength. All values are displayed as mean values.

**Table 2:** 5-yr longitudinal changes in muscle properties and adipose tissue

		Female(n=72)	Male(n=48)	Total(n=120)
Paraspinal muscle size (cm^2^)	Baseline	35.3±5.2	45.8±8.8	39.5±8.6
	5-year follow-up	34.3±5.8	45.3±8.9	38.7±9.0
	P value	<0.01	0.19	<0.01
Paraspinal muscle density (HU)	Baseline	31.5±5.3	38.2±5.9	34.2±6.4
	5-year follow-up	27.3±5.6	33.5±6.2	29.8±6.6
	P value	<0.01	<0.01	<0.01
Psoas major muscle size (cm^2^)	Baseline	14.2±2.8	22.4±3.7	17.5±5.2
	5-year follow-up	13.2±2.7	20.2±3.8	16.0±4.7
	P value	<0.01	<0.01	<0.01
Psoas major muscle density (HU)	Baseline	38.4±3.4	41.0±4.5	39.4±4.1
	5-year follow-up	34.6±3.8	36.8±4.6	35.5±4.3
	P value	<0.01	<0.01	<0.01
Extensors muscle size (cm^2^)	Baseline	38.4±6.5	44.3±9.0	40.8±8.1
	5-year follow-up	34.2±6.0	41.0±8.2	37.0±7.7
	P value	<0.01	<0.01	<0.01
Extensors muscle density (HU)	Baseline	32.0±6.0	37.9±6.3	34.4±6.8
	5-year follow-up	28.2±6.0	34.6±6.6	30.8±7.0
	P value	<0.01	<0.01	<0.01
External rotators muscle size (cm^2^)	Baseline	27.5±3.3	34.9±4.8	30.4±5.4
	5-year follow-up	26.0±3.0	33.5±5.0	29.0±5.4
	P value	<0.01	<0.01	<0.01
External rotators muscle density (HU)	Baseline	40.5±3.4	44.5±3.5	42.1±4.0
	5-year follow-up	37.5±3.7	40.7±3.4	38.8±3.9
	P value	<0.01	<0.01	<0.01
Adductors muscle size (cm^2^)	Baseline	6.8±1.3	9.0±1.9	7.7±1.9
	5-year follow-up	6.0±1.0	7.7±1.7	6.7±1.5
	P value	<0.01	<0.01	<0.01
Adductors muscle density (HU)	Baseline	36.0±5.1	41.4±5.1	38.2±5.8
	5-year follow-up	36.0±4.6	40.1±5.1	37.7±5.2
	P value	0.99	<0.01	<0.01
Flexors muscle size (cm^2^)	Baseline	11.8±1.7	15.9±2.4	13.4±2.8
	5-year follow-up	10.7±1.4	14.4±2.2	12.2±2.5
	P value	<0.01	<0.01	<0.01
Flexors muscle density (HU)	Baseline	45.0±3.7	45.7±4.0	45.2±3.8
	5-year follow-up	44.2±3.9	44.3±3.7	44.2±3.8
	P value	<0.01	<0.01	<0.01
Abductors muscle size (cm^2^)	Baseline	13.9±2.8	18.4±3.3	15.7±3.7
	5-year follow-up	13.0±2.6	17.3±3.5	14.8±3.6
	P value	<0.01	<0.01	<0.01
Abductors muscle density (HU)	Baseline	31.9±4.9	38.4±3.7	34.5±5.5
	5-year follow-up	30.4±5.2	36.0±4.1	32.6±5.5
	P value	<0.01	<0.01	<0.01
Anterior compartment of thigh muscle size (cm^2^)	Baseline	47.0±6.2	63.3±10.5	53.5±11.4
	5-year follow-up	39.5±5.6	57.6±9.7	46.7±11.6
	P value	<0.01	<0.01	<0.01
Anterior compartment of thigh muscle density (HU)	Baseline	47.4±4.1	50.0±3.5	48.4±4.0
	5-year follow-up	45.5±4.0	49.1±3.5	47.0±4.2
	P value	<0.01	<0.01	<0.01
Medial compartment of thigh muscle size (cm^2^)	Baseline	37.2±4.8	45.0±7.4	40.3±7.1
	5-year follow-up	36.5±5.4	44.1±6.9	39.5±7.1
	P value	0.05	0.15	0.02
Medial compartment of thigh muscle density (HU)	Baseline	43.0±4.5	44.2±4.3	43.5±4.4
	5-year follow-up	40.9±4.4	41.6±3.9	41.2±4.2
	P value	<0.01	<0.01	<0.01
Posterior compartment of thigh muscle size (cm^2^)	Baseline	16.3±3.5	20.6±4.8	18.1±4.6
	5-year follow-up	13.6±3.2	18.1±4.6	15.4±4.4
	P value	<0.01	<0.01	<0.01
Posterior compartment of thigh muscle density (HU)	Baseline	38.5±5.6	41.6±4.8	39.8±5.5
	5-year follow-up	30.7±5.7	33.5±6.4	31.8±6.1
	P value	<0.01	<0.01	<0.01
SAA at the L3/4 level (cm^2^)	Baseline	148.8±49.3	109.9±42.5	133.2±50.3
	5-year follow-up	165.4±54.1	116.5±45.2	145.8±55.9
	P value	<0.01	<0.01	<0.01
VAA at the L3/4 level (cm^2^)	Baseline	180.1±53.2	225.9±74.4	198.4±66.2
	5-year follow-up	202.0±56.8	236.7±73.4	215.9±65.9
	P value	<0.01	<0.01	<0.01
TAA at the L3/4 level (cm^2^)	Baseline	328.9±85.2	335.8±102.4	331.1±92.1
	5-year follow-up	367.5±95.1	353.2±100.8	361.8±97.2
	P value	<0.01	<0.01	<0.01

SAA, subcutaneous adipose area; VAA, visceral adipose area; TAA, total adipose area.

**Table 3:** **Key 5-Year Changes in Muscle and Adipose Tissue**

Parameter	Female	Male	Total
**Muscle Size (% decline)**			
- Anterior thigh	-15.8%	-8.8%	-12.3%*
- Posterior thigh	-16.4%	-12.4%	-14.4%*
- Psoas major	-6.6%*	-10.1%	-8.3%*
**Muscle Density (% decline)**			
- Extensors	-12.2%*	-9.0%*	-10.6%*
- Posterior thigh	-20.3%*	-19.6%*	-20.0%
**Adipose Tissue (% increase)**			
- SAA	+ 11.9%	+ 6.4%	+ 9.2%*
- VAA	+ 13.6%*	+ 5.7%	+ 9.7%*

*P* < 0.05 for all changes. SAA: Subcutaneous Adipose Area; VAA: Visceral Adipose Area. All values are displayed as mean values.

**Table 4:** Gender-Specific Associations: Muscle Atrophy and Functional Decline

Muscle Group	Female (OR for HGS decline)	Female (OR for HGS decline)	Female (OR for TUG decline)
Anterior thigh size	2.01* (1.10–3.67)	1.96 (0.88–4.36)	2.21* (1.16–4.24)
Psoas major size	1.90* (1.11, 3.28)	2.47 (0.90, 6.75)	1.39 (0.81, 2.37)
Psoas major density	1.47 (0.89–2.42)	0.37* (0.15–0.95)	1.64 (0.93–2.90)

*Odds Ratios (95% CI) adjusted for age, BMI, and diabetes. P<0.05.

### Association of changes in muscle with changes in muscle performance

Table 4 shows the gender-specific associations between muscle atrophy and functional decline. In females, each decrease (per cm^2^) of anterior compartment of thigh muscle size (OR:2.01, *P* = 0.02) and psoas major muscle size (OR:1.90, *P* = 0.02) would increase the risk of decreased HGS. Each decrease (per cm^2^) of anterior compartment of thigh muscle size would increase the risk of longer TUG (OR, 2.21; *P* = 0.02). Among males, each decrease (per HU) of psoas major muscle density (OR, 0.37; *P* = 0.04) would decrease the risk of decreased HGS. Associations between changes in body composition and changes in TUG were not significant in males. Table A2 displays the association of baseline body composition with baseline HGS and TUG. Table A3 shows the longitudinal association of changes in body composition with changes in HGS and TUG.

## Discussion

Our longitudinal study provides novel insights into the 5-year trajectory of age-related skeletal muscle atrophy, adipose tissue accumulation, and their associations with declining muscle strength and physical performance in community-dwelling older adults. Key findings include significant reductions in muscle size and density across multiple compartments (paraspinal, pelvic and thigh muscles), greater adipose tissue increase in females, and sex-specific associations between thigh muscle atrophy and declines in HGS and TUG performance. These results align with prior cross-sectional evidence of age-related sarcopenia and ectopic fat redistribution but extend them by quantifying longitudinal changes and their functional consequences.

The observed hierarchical patterns of muscle atrophy—particularly the pronounced decline in posterior thigh muscles and paraspinal muscle density—may reflect biomechanical adaptations to aging, such as postural shifts or reduced spinal stability. It is possible that age-associated changes in posture and gait patterns contribute to the greater atrophy in the posterior compartment [23]. We named these Jishuitan patterns for muscle atrophy. This information is very interesting because it tracks the same individual during the aging process and it will be helpful for making the preventative strategies for age-related atrophy.

The greater anterior thigh muscle atrophy in females, coupled with its strong association with HGS and TUG decline, underscores sex-specific vulnerabilities in muscle groups critical for daily mobility. This aligns with studies linking quadriceps atrophy to falls and disability but highlights the need for targeted interventions in women to preserve anterior compartment function [24–26]. Notably, the lack of association between paraspinal muscle changes and physical performance in males may reflect compensatory hypertrophy of trunk stabilizers, as suggested by Naruse et al. [23]. However, the absence of dietary or physical activity data limits our ability to explain inter-individual variability in atrophy rates. For instance, protein intake or resistance training could modulate muscle fiber composition (e.g., type II fiber preservation), which is critical for power-dependent tasks like TUG. Integrating accelerometry or dietary logs in future work could identify modifiable lifestyle factors to mitigate declines.

Few studies have investigated the relationship between changes in body composition and changes in muscle performance up to now. Caetano et al. reported that lower anterior compartment of thigh muscle strength was associated with increased risk of falls in older individuals [27]. Park et al. showed that physical performance correlated with lower limb muscle mass in older individuals [28]. Interestingly, our results suggested that changes in anterior and posterior compartments showed different associations with muscle strength and physical performance. Naruse et al. reported that age-related atrophy of the anterior and posterior compartment followed different trajectories over the lifespan [23]. Furthermore, skeletal muscles with different functions have different fiber distributions, which also play a critical role in muscle atrophy. In the thigh, the anterior compartment has a higher proportion of type II fibers for strong muscle contraction, while the posterior compartment has a more balanced distribution of type I and II fibers [29]. Thus, it may assume that muscle strength- and physical performance-related changes are more prominent in the anterior compartment, with a higher proportion of type II fibers that show muscle function decline during aging.

We found that SAA, VAA and TAA increased with aging. The changes in VAA are analogous to an increase in intermuscular fat, which is related to an increase in proinflammatory cytokines [30]. Fat redistributes from subcutaneous white adipose tissues depots to visceral white adipose tissues depots during and after middle age [31]. The paradoxical increase in subcutaneous adipose area (SAA) alongside visceral adipose area (VAA) contrasts with some prior reports of adipose tissue decline in advanced age, likely due to methodological differences in imaging (CT vs. DXA) [32].

The study’s limitations—modest sample size, single geographic cohort, and restricted functional assessments—highlight opportunities for expansion. Further, in these subjects we did not measure Vitamin D that could theoretically influence muscle strength, which would impact the explanations of muscle strength decline. Larger, diverse cohorts would improve generalizability and enable subgroup analyses (e.g., diabetes severity, age ≥ 80). Adding gait speed or balance tests would better assess functional decline. Longitudinal interventions, such as resistance training or nutritional supplementation, should be piloted to test whether slowing anterior thigh atrophy in females improves HGS/TUG trajectories. Clinically, our findings support using opportunistic CT-derived muscle density thresholds as early markers of functional decline. For example, the 12.2% decline in female extensor muscle density could inform targeted screening for mobility limitations. Machine learning models leveraging baseline body composition data may further refine risk prediction for falls or disability.

In conclusion, this study shows different patterns of muscle and adipose tissue aging, emphasizing sex-specific risks and the prognostic value of thigh muscle metrics. Future research should prioritize lifestyle-behavioral linkages, advanced imaging, and specific interventions to promote functional performance in older adults.

## Data Availability

No datasets were generated or analysed during the current study.

## Appendix

See Appendix tables (5, 6 and 7).

**Table 5:** 5-yr percent changes in muscle properties and adipose tissue by gender and age

	Gender			Female			Male		
	Female (*n* = 72)	Male (*n* = 48)	*P* value	Age (< 70) (*n* = 47)	Age (≥ 70) (*n* = 25)	*P* value	Age (< 70) (*n* = 30)	Age (≥ 70) (*n* = 18)	*P* value
Paraspinals muscle size (%)	-2.9 (-18.7, 8.1)	-1.0 (-11.3, 9.0)	0.09	-2.1 (-13.2, 8.1)	-4.5 (-18.7, 6.9)	0.19	-0.5 (-11.2, 9.0)	-1.8 (-11.3, 3.9)	0.42
Paraspinals muscle density (%)	-13.5 (-38.3, 4.6)	-12.5 (-25.5, -2.0)	0.48	-15.4 (-38.3, -1.4)	-10.0 (-21.8, 4.6)	0.02	-10.5 (-21.5, -2.0)	-15.8 (-25.5, -6.8)	< 0.01
Psoas major muscle size (%)	-6.6 (-24.5, 11.5)	-10.1 (-24.5, 1.5)	0.02	-3.9 (-18.8, 11.6)	-11.6 (-24.5, 6.5)	< 0.01	-9.4 (-23.1, 1.5)	-11.2 (-24.5, -1.5)	0.43
Psoas major muscle density (%)	-9.9 (-19.5, 5.7)	-10.4 (-18.4, 0.4)	0.59	-8.9 (16.6, 5.7)	-11.6 (-19.5, -1.3)	0.04	-9.2 (-15.4, 0.4)	-12.2 (-18.4, -5.2)	0.02
Extensors muscle size (%)	-10.5 (-27.9, 16.2)	-7.0 (-22.3, 8.9)	0.02	-9.5 (-24.7, 16.2)	-12.4 (-27.9, 6.6)	0.25	-5.5 (-13.9, 8.9)	-9.5 (-22.3, 0.2)	0.03
Extensors muscle density (%)	-12.2 (-26.4, 7.6)	-9.0 (-16.1, 2.1)	< 0.01	-10.7 (-24.5, 7.6)	-15.1 (-26.4, -0.8)	0.02	-8.0 (-14.4, 2.1)	-10.7 (-16.1, -5.0)	0.02
External rotators muscle size (%)	-5.1 (-23.3, 11.5)	-3.6 (-17.4, 14.3)	0.37	-5.6 (-23.3, 11.5)	-4.2 (-21.3, 8.9)	0.50	-2.4 (-17.4, 14.3)	-5.7 (-17.1, 5.4)	0.20
External rotators muscle density (%)	-7.1 (-22.4, 4.2)	-8.5 (-14.7, 1.5)	0.16	-6.6 (-20.3, 4.2)	-8.2 (-22.4, 1.6)	0.32	-7.8 (-14.3, 1.5)	-9.5 (-14.7, -2.4)	0.13
Adductors muscle size (%)	-10.8 (-28.9, 16.3)	-12.7 (-29.6, 5.2)	0.35	-9.3 (-28.9, 16.3)	-13.7 (-24.9, 3.4)	0.07	-11.0 (-29.2, 5.2)	-15.4 (-29.6, -0.4)	0.14
Adductors muscle density (%)	0.5 (-14.9, 19.1)	-3.1 (-14.6, 8.7)	< 0.01	0.6 (14.9, 19.1)	0.1 (-9.0, 7.2)	0.76	-2.4 (-10.4, 8.7)	-4.3 (-14.6, 1.3)	0.25
Flexors muscle size (%)	-8.7 (-26.5, 8.5)	-8.6 (-23.2, 9.8)	0.95	-9.1 (-26.5, 8.0)	-7.8 (-24.0, 8.5)	0.56	-7.4 (-17.8, 9.8)	-10.6 (-23.2, -2.0)	0.19
Flexors muscle density (%)	-1.8 (-11.8, 6.1)	-2.9 (-10.7, 4.9)	0.15	-1.3 (-8.9, 6.1)	-2.7 (-11.8, 5.6)	0.26	-1.3 (-6.8, 4.9)	-5.7 (-10.7, -1.7)	< 0.01
Abductors muscle size (%)	-6.0 (-23.7, 11.7)	-5.8 (-20.7, 5.8)	0.90	-5.3 (-21.3, 11.7)	-7.2 (-23.7, 10.1)	0.40	-4.9 (-15.3, 5.8)	-7.4 (-20.7, 4.8)	0.22
Abductors muscle density (%)	-4.7 (-15.8, 14.9)	-6.2 (-16.9, 4.4)	0.20	-3.0 (-15.8, 14.9)	-8.0 (-13.4, -0.5)	< 0.01	-4.7 (-13.2, 4.4)	-8.9 (-16.9, 1.5)	< 0.01
Anterior compartment of thigh muscle size (%)	-15.8 (-28.4, -0.6)	-8.8 (-22.5, 9.7)	< 0.01	-15.4 (-28.4, -0.6)	-16.3 (-26.9, -6.4)	0.61	-7.6 (-22.5, 9.7)	-10.8 (-22.2, -0.1)	0.12
Anterior compartment of thigh muscle density (%)	-4.0 (-12.1, 1.9)	-1.6 (-8.4, 6.9)	< 0.01	-3.9 (-9.9, 1.9)	-4.0 (-12.1, 0.8)	0.90	-0.8 (-6.4, 6.9)	-3.0 (-8.4, 1.1)	0.03
Medial compartment of thigh muscle size (%)	-1.9 (-17.1, 14.8)	-1.5 (-20.4, 16.2)	0.82	-1.6 (-12.9, 14.8)	-2.4 (-17.1, 13.0)	0.69	-1.3 (-20.4, 16.2)	-1.8 (-13.4, 11.0)	0.87
Medial compartment of thigh muscle density (%)	-4.8 (-16.8, 8.4)	-5.7 (-19.3, 9.1)	0.50	-3.9 (-10.3, 8.4)	-6.6 (-16.8, 2.2)	0.04	-5.5 (-19.3, 9.1)	-5.9 (-16.7, 6.3)	0.88
Posterior compartment of thigh muscle size (%)	-16.4 (-34.6, 17.6)	-12.4 (-25.3, -1.3)	0.02	-15.6 (-34.6, 17.6)	-17.9 (-33.7, -0.4)	0.37	-12.0 (-21.4, -1.3)	-13.1 (-25.3, -1.4)	0.56
Posterior compartment of thigh muscle density (%)	-20.3 (-45.1, 2.7)	-19.6 (-41.6, 6.7)	0.76	-17.8 (-33.5, 2.7)	-24.9 (-45.1, -4.4)	0.02	-16.8 (-34.3, 6.7)	-24.4 (-41.6, -10.4)	0.02
SAA (%)	11.9 (-9.9, 49.8)	6.4 (-6.7, 18.0)	< 0.01	10.1 (-9.9, 47.8)	15.2 (0.3, 49.8)	0.14	5.8 (-6.7, 18.0)	7.6 (-0.9, 16.1)	0.37
VAA (%)	13.6 (-15.3, 59.4)	5.7 (-5.9, 19.7)	< 0.01	16.3 (-10.2, 59.4)	8.5 (-15.3, 26.9)	0.03	6.0 (-4.5, 19.7)	5.0 (-5.9, 13.2)	0.64
TAA (%)	12.2 (-9.2, 40.3)	5.8 (-5.1, 19.1)	< 0.01	12.7 (-9.2, 40.3)	11.1 (-4.1, 32.4)	0.58	6.0 (-5.1, 19.1)	5.6 (-4.6, 12.1)	0.86

SAA, subcutaneous adipose area; VAA, visceral adipose area; TAA, total adipose area.

**Table 6:** Association between baseline body composition and baseline HGS, baseline TUG, adjusted for baseline age, height, weight and type 2 diabetes

	Association with baseline HGS				Association with baseline TUG			
	Female (*n* = 72)		Male (*n* = 48)		Female (*n* = 72)		Male (*n* = 48)	
Baseline muscle properties	OR (95% CI)	P value	OR (95% CI)	P value	OR (95% CI)	P value	OR (95% CI)	P value
Paraspinals muscle size	1.28 (0.50, 3.29)	0.61	0.62 (0.24, 1.57)	0.31	1.29 (0.50, 3.34)	0.59	1.08 (0.53, 2.19)	0.83
Paraspinals muscle density	1.22 (0.62, 2.38)	0.56	0.68 (0.29, 1.58)	0.37	0.75 (0.37, 1.50)	0.41	1.24 (0.57, 2.70)	0.60
Psoas major muscle size	1.32 (0.50, 3.53)	0.58	1.54 (0.45, 5.25)	0.49	1.78 (0.67, 4.71)	0.25	1.61 (0.62, 4.18)	0.33
Psoas major muscle density	1.41 (0.75, 2.62)	0.29	1.60 (0.65, 3.92)	0.31	0.50 (0.24, 1.05)	0.07	0.77 (0.39, 1.54)	0.46
Extensors muscle size	1.26 (0.63, 2.52)	0.51	0.74 (0.35, 1.60)	0.45	1.77 (0.82, 3.79)	0.15	0.97 (0.49, 1.89)	0.92
Extensors muscle density	2.66 (1.22, 5.80)	0.01	0.51 (0.21, 1.25)	0.14	1.04 (0.53, 2.02)	0.92	1.29 (0.66, 2.52)	0.46
External rotators muscle size	0.68 (0.28, 1.68)	0.40	1.17 (0.50, 2.76)	0.71	1.42 (0.57, 3.53)	0.45	0.73 (0.35, 1.53)	0.40
External rotators muscle density	0.81 (0.43, 1.52)	0.50	3.57 (0.91, 14.02)	0.07	1.15 (0.61, 2.18)	0.66	0.82 (0.36, 1.88)	0.64
Adductors muscle size	2.00 (0.89, 4.50)	0.10	1.91 (0.89, 4.07)	0.10	1.78 (0.80, 3.97)	0.16	0.97 (0.52, 1.83)	0.92
Adductors muscle density	1.57 (0.84, 2.96)	0.16	1.14 (0.51, 2.53)	0.75	1.07 (0.58, 1.98)	0.83	0.93 (0.47, 1.83)	0.82
Flexors muscle size	2.21 (0.83, 5.86)	0.11	0.88 (0.31, 2.50)	0.81	1.23 (0.49, 3.09)	0.66	0.64 (0.25, 1.61)	0.34
Flexors muscle density	1.13 (0.66, 1.95)	0.65	0.99 (0.45, 2.17)	0.97	0.81 (0.47, 1.41)	0.46	1.07 (0.55, 2.06)	0.85
Abductors muscle size	0.93 (0.46, 1.90)	0.85	1.19 (0.46, 3.03)	0.72	1.83 (0.87, 3.85)	0.11	1.31 (0.59, 2.91)	0.51
Abductors muscle density	2.59 (1.26, 5.29)	< 0.01	1.16 (0.38, 3.53)	0.80	1.33 (0.71, 2.50)	0.37	1.62 (0.60, 4.39)	0.35
Anterior compartment of thigh muscle size	1.64 (0.51, 5.30)	0.41	1.34 (0.56, 3.20)	0.51	3.51 (0.98, 12.56)	0.05	1.08 (0.51, 2.26)	0.85
Anterior compartment of thigh muscle density	1.29 (0.72, 2.28)	0.39	1.16 (0.42, 3.26)	0.77	0.80 (0.44, 1.47)	0.47	0.78 (0.35, 1.74)	0.55
Medial compartment of thigh muscle size	1.01 (0.48, 2.10)	0.99	0.76 (0.35, 1.62)	0.47	1.01 (0.47, 2.17)	0.98	0.78 (0.42, 1.45)	0.44
Medial compartment of thigh muscle density	1.31 (0.78, 2.21)	0.31	0.75 (0.32, 1.73)	0.50	0.96 (0.57, 1.63)	0.87	1.46 (0.71, 3.01)	0.30
Posterior compartment of thigh muscle size	0.97 (0.47, 2.01)	0.94	0.95 (0.48, 1.89)	0.88	1.56 (0.73, 3.32)	0.25	1.17 (0.65, 2.10)	0.60
Posterior compartment of thigh muscle density	2.30 (1.24, 4.27)	< 0.01	0.43 (0.15, 1.26)	0.12	1.07 (0.61, 1.87)	0.82	1.41 (0.65, 3.02)	0.38
Baseline adipose tissue								
SAA	0.60 (0.29, 1.25)	0.17	2.32 (0.72, 7.45)	0.16	1.28 (0.62, 2.62)	0.50	0.64 (0.25, 1.62)	0.34
VAA	0.88 (0.41, 1.86)	0.73	0.36 (0.09, 1.42)	0.14	0.73 (0.34, 1.57)	0.42	0.37 (0.14, 1.00)	0.05
TAA	0.62 (0.26, 1.44)	0.26	0.86 (0.28, 2.63)	0.80	0.92 (0.41, 2.07)	0.83	0.34 (0.12, 0.99)	0.04

BMI, body mass index; TUG, the Timed Up and Go test; HGS, handgrip strength; CI, confidence interval; SAA, subcutaneous adipose area; VAA, visceral adipose area; TAA, total adipose area.

**Table 7:** Longitudinal association between percent changes in body composition and percent changes in HGS, in TUG, adjusted for baseline age, height, weight and type 2 diabetes

	Association with percent changes in HGS				Association with percent changes in TUG			
	Female (*n* = 72)		Male (*n* = 48)		Female (*n* = 72)		Male (*n* = 48)	
Percent changes in muscle properties	OR (95% CI)	P value	OR (95% CI)	P value	OR (95% CI)	P value	OR (95% CI)	P value
Paraspinals muscle size	1.25 (0.77, 2.04)	0.37	1.51 (0.68, 3.32)	0.31	0.99 (0.59, 1.66)	0.96	1.85 (0.85, 4.06)	0.12
Paraspinals muscle density	1.50 (0.93, 2.41)	0.09	0.96 (0.37, 2.50)	0.94	1.49 (0.90, 2.45)	0.12	1.56 (0.62, 3.96)	0.35
Psoas major muscle size	1.90 (1.11, 3.28)	0.02	2.47 (0.90, 6.75)	0.08	1.39 (0.81, 2.37)	0.23	1.34 (0.60, 3.02)	0.47
Psoas major muscle density	1.47 (0.89, 2.42)	0.14	0.37 (0.15, 0.95)	0.04	1.64 (0.93, 2.90)	0.09	1.22 (0.58, 2.55)	0.60
Extensors muscle size	1.13 (0.71, 1.80)	0.61	1.10 (0.43, 2.84)	0.84	0.87 (0.52, 1.46)	0.60	1.26 (0.52, 3.07)	0.61
Extensors muscle density	1.19 (0.75, 1.88)	0.46	0.78 (0.24, 2.52)	0.67	0.99 (0.59, 1.64)	0.96	1.08 (0.36, 3.29)	0.89
External rotators muscle size	1.10 (0.66, 1.85)	0.71	1.30 (0.62, 2.74)	0.49	1.07 (0.61, 1.88)	0.82	1.55 (0.77, 3.12)	0.22
External rotators muscle density	1.03 (0.67, 1.58)	0.88	0.60 (0.22, 1.63)	0.32	1.62 (0.98, 2.69)	0.06	0.64 (0.24, 1.74)	0.38
Adductors muscle size	1.27 (0.78, 2.09)	0.34	1.43 (0.66, 3.08)	0.36	1.01 (0.60, 1.69)	0.99	1.01 (0.48, 2.10)	0.99
Adductors muscle density	0.85 (0.50, 1.43)	0.54	1.19 (0.52, 2.72)	0.69	1.09 (0.63, 1.87)	0.76	2.08 (0.84, 5.14)	0.11
Flexors muscle size	1.25 (0.76, 2.06)	0.38	2.04 (0.93, 4.50)	0.08	0.98 (0.58, 1.67)	0.95	0.82 (0.42, 1.61)	0.57
Flexors muscle density	1.27 (0.78, 2.05)	0.34	1.15 (0.48, 2.78)	0.75	1.28 (0.76, 2.17)	0.35	1.17 (0.48, 2.86)	0.74
Abductors muscle size	1.98 (1.17, 3.34)	0.01	1.91 (0.79, 4.58)	0.15	1.33 (0.80, 2.19)	0.27	0.94 (0.43, 2.06)	0.88
Abductors muscle density	1.31 (0.78, 2.22)	0.31	0.65 (0.27, 1.59)	0.34	0.91 (0.54, 1.52)	0.71	0.75 (0.32, 1.77)	0.51
Anterior compartment of thigh muscle size	2.01 (1.10, 3.67)	0.02	1.96 (0.88, 4.36)	0.10	2.21 (1.16, 4.24)	0.02	1.07 (0.53, 2.13)	0.85
Anterior compartment of thigh muscle density	0.96 (0.57, 1.62)	0.87	0.98 (0.46, 2.10)	0.96	0.81 (0.46, 1.43)	0.47	1.48 (0.67, 3.24)	0.33
Medial compartment of thigh muscle size	0.96 (0.55, 1.66)	0.88	1.81 (0.90, 3.63)	0.10	1.23 (0.67, 2.25)	0.50	0.88 (0.48, 1.60)	0.67
Medial compartment of thigh muscle density	0.87 (0.49, 1.55)	0.64	1.12 (0.63, 2.00)	0.70	1.14 (0.61, 2.12)	0.68	0.81 (0.46, 1.42)	0.46
Posterior compartment of thigh muscle size	0.63 (0.39, 1.01)	0.06	1.02 (0.37, 2.79)	0.97	0.92 (0.59, 1.46)	0.73	0.82 (0.30, 2.22)	0.69
Posterior compartment of thigh muscle density	0.57 (0.32, 1.02)	0.06	1.70 (0.84, 3.43)	0.14	0.78 (0.43, 1.42)	0.42	0.76 (0.39, 1.49)	0.43
Percent changes in adipose tissue								
SAA	0.88 (0.56, 1.37)	0.57	0.83 (0.27, 2.56)	0.75	0.71 (0.42, 1.18)	0.19	0.80 (0.26, 2.42)	0.69
VAA	0.81 (0.51, 1.27)	0.35	1.19 (0.35, 4.01)	0.78	0.45 (0.25, 0.81)	< 0.01	0.41 (0.11, 1.49)	0.18
TAA	0.90 (0.57, 1.41)	0.64	1.12 (0.35, 3.54)	0.85	0.48 (0.27, 0.86)	0.01	0.51 (0.15, 1.71)	0.28

BMI, body mass index; TUG, the Timed Up and Go test; HGS, handgrip strength; CI, confidence interval; SAA, subcutaneous adipose area; VAA, visceral adipose area; TAA, total adipose area.
